# The Impact of Perceptual Load and Distractors’ Perceptual Grouping on Visual Search in ASD

**DOI:** 10.3390/bs16010080

**Published:** 2026-01-07

**Authors:** Wenyi Shen, Yijie Huang, Lin Zhang, Shimin Fu

**Affiliations:** 1 Department of Psychology and Center for Brain and Cognitive Sciences, School of Education, Guangzhou University, Guangzhou 510006, China; 2 Department of Psychology, The University of Oklahoma, Norman, OK 73019, USA; 3 School of Educational Sciences, Guangdong Polytechnic Normal University, Guangzhou 510665, China

**Keywords:** adults with high-functioning autism spectrum disorder, visual search, perceptual load, perceptual grouping, asymmetry

## Abstract

This study examined potential visual search advantages in individuals with autism spectrum disorder (ASD) and explored the roles of distractor grouping and perceptual load by comparing their performance with that of typically developing (TD) controls. Participants were required to search for large or small targets under two levels of perceptual load, with distractors being either large or small. The results showed the following: (1) Search speed in the ASD group was slower than that of the TD group. (2) The effect of distractor grouping was stronger in the Target–Nontarget (T-N) size-inconsistent condition than in the consistent condition. Both groups showed a T-N size-consistency effect—response speeds in the T-N size-inconsistent condition were faster, indicating that distractor grouping improves search efficiency. (3) Under high load, the TD group exhibited a stronger T-N size-consistency effect than the ASD group, whereas no significant difference was observed under low load. This suggests that distractor grouping in the ASD group is less effective than in TD participants under high load. (4) Under the T-N size-inconsistent condition, participants with ASD detected small targets faster under low load, whereas TD participants detected large targets faster under high load. This indicates that distractor grouping facilitates visual search in ASD under low load. Both groups focus more on targets under high load. In conclusion, although ASD shows no search advantage, improving distractor grouping can speed up target search. Nevertheless, under high load, distractor grouping in individuals with ASD is weaker than in TD individuals, consistent with the weak central coherence theory. Additionally, ASD displays size asymmetry that is influenced by load, with distractor grouping aiding target detection in low load and reducing distractor processing under high load.

## 1. Introduction

According to the fifth edition of the Diagnostic and Statistical Manual of Mental Disorders (DSM-5), autism spectrum disorder (ASD) is characterized as a neurodevelopmental disorder marked by two core symptom domains: deficits in social communication and social interaction, and the presence of restricted, repetitive patterns of behavior and interests (American Psychiatric Association, 2013). These primary symptoms manifest in attentional content and strategies (Grubb et al., 2013). Specifically, impairments in social communication lead individuals with ASD to overlook social cues—such as facial expressions—while exhibiting an attentional bias toward non-social stimuli, such as geometric figures and symbols. Concurrently, restricted and repetitive behaviors and interests are associated with alterations in visual search strategies, often favoring local processing while demonstrating difficulties in global integration (Masedu et al., 2022; Vasudeva & Eric, 2017).

Individuals with ASD are of particular interest to researchers due to their distinctive selective attention, especially their unique visual search patterns (Allenmark et al., 2021; Van de Cruys et al., 2021). Visual search paradigms serve as an effective method for examining two core aspects of attentional and perceptual grouping. On the one hand, it can examine the attention efficiency of target search from distractors (Treisman & Gelade, 1980; Wolfe, 2021). On the other hand, it examines the influence of distractor similarity-based perceptual grouping on search performance (Duncan & Humphreys, 1989). Notably, ASD may exhibit differences in attentional and perceptual processing compared to typically developing (TD) populations (Burns et al., 2017; DuBois et al., 2017; Dellapiazza et al., 2018).

### 1.1. The Advantages of Visual Search in ASD

The debate regarding whether ASD demonstrates accelerated visual search performance compared to TD remains unresolved. Some empirical evidence suggests that ASD confers an advantage in visual search paradigms, particularly in conjunction search tasks. When targets are distinguished from distractors through the integration of multiple features, ASD participants exhibit superior search efficiency relative to TD (Plaisted et al., 1998; Amrita et al., 2014; Joseph et al., 2009; Shirama et al., 2017). Conversely, other studies report comparable or even diminished visual search capabilities in ASD, with some indicating deficits relative to normative benchmarks (Constable, 2010; Keehn & Joseph, 2016; Marciano et al., 2021).

The underlying factors contributing to this inconsistency stem primarily from two sources. First, the heterogeneity inherent within ASD significantly influences outcomes (Lindor et al., 2018). Second, the modulation of visual search speed by task complexity plays a critical role (Zhong & Chen, 2022). As task demands escalate from efficient feature-based searches—where attention is rapidly captured by salient target features—to less efficient search modalities requiring serial attentional shifts, and ultimately to tasks necessitating detailed stimulus analysis to differentiate targets from distractors, the difficulty level increases (Joseph et al., 2009). In low-difficulty, single-feature search tasks—such as locating a “0” within an “O” based solely on shape (Constable, 2010), or identifying an upright ‘T’ among rotated variants (Keehn et al., 2013)—the advantage in ASD is negligible or absent. However, in more complex tasks involving multiple feature conjunctions or categorical distinctions, individuals with ASD generally demonstrate slower search times compared to TD individuals. The former tasks have targets that differ from distractors across several features, and target stimuli vary across trials (Keehn & Joseph, 2016). The latter tasks demand the integration of multiple features or the categorization of stimuli, such as distinguishing images of animals among numerous artificial objects (Doherty et al., 2018).

### 1.2. Perceptual Load and Perceptual Grouping in Visual Search in ASD

Perceptual load is a critical factor influencing attentional resource allocation during visual search. The perceptual load theory (Lavie, 2005) posits that an individual’s perceptual capacity is limited and that all stimuli are processed automatically until resources are exhausted. Under low perceptual load, surplus resources are available for processing distractors; conversely, as perceptual load increases, the resources allocated to distractor processing diminish (Cheng et al., 2023; Murphy et al., 2016). In visual search tasks, prior research predominantly manipulated set size (Lavie & Cox, 1997) or increased stimulus features of distractors and targets, such as orientation (Peng & Huang, 2018), color (Zhong & Chen, 2022), and size (Shen et al., 2021), to modulate perceptual load. However, these approaches often overlook the fact that independently altering distractor and target features can induce different distractor grouping effects, which in turn influence search efficiency (Palmer & Beck, 2007). Specifically, when selecting a target among distractors, perceptual grouping based on shared features or spatial relationships—such as similar shape, size, or close proximity—facilitates the formation of perceptual units or basic elements, thereby accelerating search performance in TD individuals (Horstmann et al., 2010; Vecera & Behrmann, 2001). Additionally, some studies suggest that when targets are more salient than distractors, similar distractors are processed collectively, easing perceptual grouping. In such cases, individuals do not engage in strictly serial processing but instead perform parallel selection and suppression of distractors, resulting in reduced overall reaction time (RT) (Brooks et al., 2017; Wagemans et al., 2012).

However, most studies suggest that ASD exhibits atypical perceptual grouping abilities, primarily characterized by an imbalance between local and global processing (Koldewyn et al., 2013). Specifically, individuals with ASD tend to demonstrate superior processing of local details accompanied by deficits in global information integration (Nilsson et al., 2018). Nonetheless, there remains insufficient evidence to determine whether individuals with ASD process more local information than TD individuals or whether they can achieve the same level of accuracy in global processing (Van der Hallen et al., 2015, 2019).

### 1.3. Factors Influencing Asymmetry in Visual Search

The prioritization and efficiency in searching for specific target features, such as “presence” or “salience” or differences in processing speed of interference (Ueda et al., 2015, 2023), may lead to asymmetries in visual search phenomena. Search asymmetry refers to the phenomenon where, in visual search tasks involving features with different properties, the search speed varies depending on whether the feature functions as the target or the distractor (Neisser, 1963; Zhang & Onyper, 2020). Classic studies demonstrating this include shape feature differences such as closed versus open circles (Treisman & Gormican, 1988), and “Q” versus “O” (Lv, 2011), which confirm the existence of asymmetry in visual processing. Subsequent research has further identified asymmetries based on differences in orientation (Russell et al., 2016), size (Shen et al., 2021), and semantic information (Shen et al., 2024).

Existing studies suggest that perceptual load influences the asymmetry observed in visual search among TD individuals. Shen et al. (2021) tasked participants with feature searches involving size stimuli as distractors and targets within set sizes of 6 and 12, using stimuli from various categories. Results showed that in a larger set size, large targets were found more quickly than small ones, whereas no significant difference was observed in a small set size. A possible explanation is that, under low perceptual load, search speed in TD individuals is generally rapid, with no significant difference between large and small targets. Conversely, under high perceptual load, attentional resources are limited and more focused on target stimuli, making larger stimuli more salient as targets (Arcangelo et al., 2020; Wühr & Seegelke, 2018), thus leading to significantly faster processing speeds. However, given the atypical perceptual grouping in ASD individuals, whether similar size asymmetry occurs during visual search and whether the size asymmetry is modulated by perceptual load remains an area lacking empirical investigation.

In summary, referencing Shen et al. (2021), we utilized multiple stimulus categories, with distractors and targets randomly presented at various locations, and manipulated perceptual load through two set sizes (6 and 12). Participants are required to search for large or small targets that differ in shape from distractors. The Target–Nontarget (T-N) size-consistency effect can separate distractors from targets, reflecting the role of distractors’ perceptual grouping in visual search. Thus, the main objectives of this study are as follows: (1) to examine whether ASD individuals demonstrated search advantage in feature search tasks; (2) to investigate how distractor grouping affects visual search in ASD; (3) to compare the differences in distractor grouping between two groups under different perceptual loads; and (4) to analyze the size asymmetry between two groups. Based on the above, the hypotheses are as follows: (1) The advantage observed in ASD may be influenced by task difficulty. This search task was more difficult than a simpler single-feature search task, so it could potentially reveal an advantage in ASD. (2) Distractor grouping facilitates faster visual search in both groups. (3) Differences in distractor grouping between ASD and TD are apparent under varying perceptual loads. (4) The size asymmetry varies between ASD and TD groups across different perceptual loads.

## 2. Materials and Methods

### 2.1. Participants

We determined a target sample size of 48 using G*Power 3.1.9.7 (Faul et al., 2007) with a moderate effect size (*f* = 0.25) for sample size estimation (α = 0.05, 1 − β = 0.95). Ultimately, 48 participants were recruited and distributed across the two groups. A total of 49 eligible right-handed participants with normal or corrected-to-normal vision were recruited through universities and community organizations. The ASD group consisted of 24 individuals who had received clinical diagnoses during childhood from the Department of Child Development or Psychiatry in tertiary hospitals. The diagnosis satisfies the requirements of the DSM-IV, ICD-10, DSM-V, and other professional diagnostic standards. None of the participants had a history of major psychiatric illness (schizophrenia, bipolar disorder, or depressive disorder) or severe physical and neurological conditions (e.g., epilepsy). To gather information on current symptoms, trained examiners used the Autism Diagnostic Interview, Revised Version (ADI-R). For each diagnostic criterion, a standard primary question was asked, followed by questions to clarify whether the participant met the criteria of the given item. This semi-structured interview has been used for diagnostic classification in previous studies (Spek et al., 2010). The TD group included 25 individuals with no significant differences in overall IQ, age, and gender compared to the ASD group (*ps* > 0.05). Arithmetic is related to mathematical computing ability and working memory. Encoding is more responsive to the processing speed of short-term memory (Cui et al., 2017). As the current research focuses on visual search ability, these two sub-dimensions are unlikely to constitute confounding factors. Participant details are presented in Table 1. All participants voluntarily participated, provided informed consent, and received compensation.

### 2.2. Design

A four-factor mixed design was employed: 2 (Group: TD vs. ASD) × 2 (Set Size: 6 vs. 12) × 2 target size (large vs. small) × 2 T-N size-consistency (consistent vs. inconsistent), where the group is the between-subjects variable and others are within-subject variables. The dependent variables are the RT and accuracy in a target present/absent task.

### 2.3. Materials

The experiment included 192 trials for the target-present condition. These stimuli were categorized into four conditions: large target-small distractor, small target-large distractor, large target-large distractor, and small target-small distractor. The search target possesses a unique shape feature, with only one such target per trial, and the number of distractor stimuli is either five or eleven. In target-absent conditions, 128 stimuli were selected, divided into two categories: large and small stimuli. All stimuli were equally drawn from four experimental categories: special symbols (e.g., ○), mathematical units (e.g., &), numbers (e.g., 8), and letters (e.g., H). The experimental materials are not repeated in the target-present and target-absent conditions. Each search target had a distinct shape, appearing only once per page, alongside 5 or 11 interfering items. The visual angles for small stimuli were 2.30 × 1.73°, and for large stimuli, 4.60 × 3.45°.

### 2.4. Procedure

**Figure 1:**
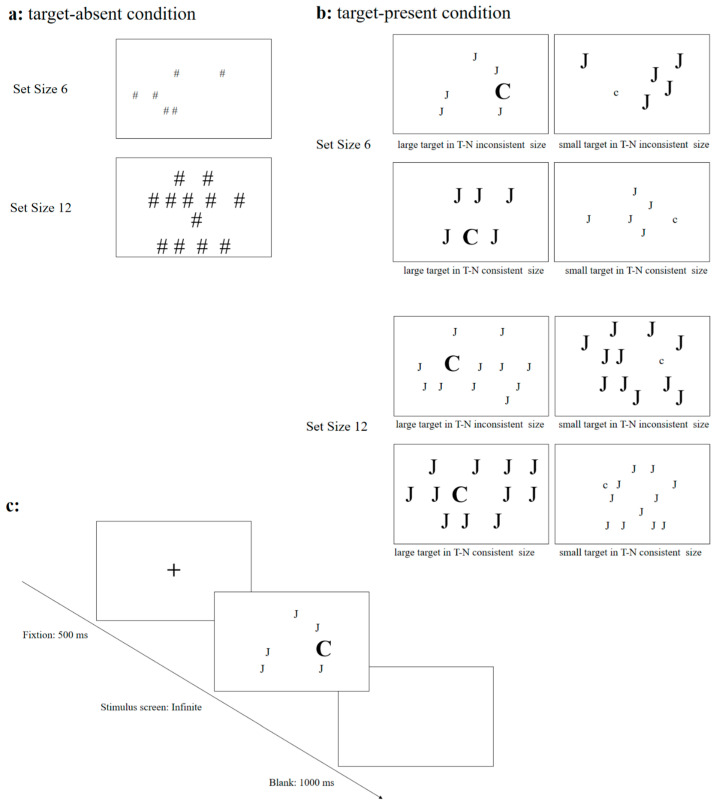
Schematic diagram of the experimental procedure and conditions: panel (**a**) depicts the non-target conditions, which constitute one-third of the total trials and serve solely to ensure participants’ attentive engagement. Panel (**b**) illustrates the target conditions. In T-N size-inconsistency, both the size and shape of the distractor differ from the target, resulting in a stronger distractor grouping compared to the T-N size-consistency, which only has a shape difference between distractor and target. Panel (**c**) presents the experimental flowchart.

The experiment was conducted in a noise-free behavioral laboratory. The procedure was programmed and executed using E-Prime 2.0.10, with stimuli presented on a 27-inch LCD monitor (resolution 1024 × 768, refresh rate 60 Hz). Each participant was tested individually, maintaining a constant viewing distance of approximately 60 cm from the monitor. Initially, a fixation cross of 500 ms was displayed at the screen’s center. Following this, the stimulus interface appeared, prompting participants to determine the presence of the target. If detected, they pressed the “J” key; if not, the “F” key. After the stimulus screen vanished, a blank screen was displayed for 1000 ms (Figure 1). The study comprises 320 trials along with 10 practice trials. Participants were uninformed about the experiment’s purpose beforehand and were tasked with swift and accurate decision-making. After completing the experiment, participants received a specific reward. The entire procedure lasted around 20 min.

## 3. Results

All participants demonstrated accuracy rates above 85% under the target condition, with 88% exceeding 95%. Mean accuracy rates exceeded 96%, with all conditions surpassing 95%. This high accuracy likely indicates a ceiling effect; therefore, accuracy data were used primarily to confirm participant engagement and were not further interpreted. The RT outliers defined as ± 2 SDs from the individual’s mean was excluded (Pinkham et al., 2010), accounting for 7.7% of the total data. Repeated measures ANOVA was conducted on the RT for both the target-absent and target-present conditions. If the spherical assumption was violated, the Greenhouse–Geisser correction was applied. All analysis were conducted utilizing the SPSS 25.0 software.

For the target-absent condition, a 2 (group: TD vs. ASD) × 2 (set size: 6 vs. 12) repeated measures ANOVA revealed no significant main effect of group, *F* (1, 47) = 0.01, *p* = 0.99, *η*^2^ = 0.01, indicating that the TD and ASD groups did not differ significantly in their response speeds for target-absent trials, thus ruling out manual response speed (button press or touchscreen RT) as a confounding factor influencing search efficiency. A significant main effect of set size was observed, *F* (1, 47) = 99.50, *p* < 0.001, *η*^2^ = 0.68, with faster RT for a small set size. The interaction between group and set size was also significant, *F* (1, 47) = 12.20, *p* = 0.001, *η^2^* = 0.21, with the TD group exhibiting a larger difference in the RT between small and large sets (242 ± 52 ms vs. 503 ± 53 ms) compared to the ASD group.

For the target-present condition (Table 2), a 2 (group: TD vs. ASD) × 2 (set size: 6 vs. 12) × 2 (target size: large vs. small) × 2 (T-N size-consistency: consistent vs. inconsistent) repeated measures ANOVA showed a significant main effect of group, *F* (1, 47) = 7.25, *p* = 0.01, *η*^2^ = 0.13, with the TD group responding faster than the ASD group (877 ± 62 ms vs. 1114 ± 63 ms). The main effect of set size was also significant, *F* (1, 47) = 16.41, *p* < 0.001, *η*^2^ = 0.26, with faster responses in small sets than large sets (976 ± 42 vs. 1015 ± 46 ms). A significant main effect of target size was found, *F* (1, 47) = 79.18, *p* < 0.001, *η*^2^ = 0.63, with larger targets eliciting faster responses than small targets (951 ± 43 ms vs. 1039 ± 45 ms). The main effect of T-N size-consistency was significant, *F* (1, 47) = 169.37, *p* < 0.001, *η*^2^ = 0.78, and the RT in the T-N size-inconsistent condition was significantly faster than in the T-N size-consistent condition (943 ± 43 ms vs. 1047 ± 46 ms). There is a significant interaction between target size and set size, *F* (1, 47) = 12.76, *p* = 0.001, *η*^2^ = 0.21, indicating greater efficiency differences between set sizes when searching for small targets than large targets. Specifically, the RT for small targets was faster at the small set size compared to the large set size (995 ± 40 ms vs. 1082 ± 52 ms, *p* < 0.001). However, when searching for a large target, there was no significant difference between the large and small set sizes (947 ± 41 ms vs. 956 ± 46 ms, *p* = 0.501). An interaction between target size and T-N size-consistency was significant, *F* (1, 47) = 39.50, *p* < 0.001, *η^2^* = 0.46, showing that the T-N size-consistency effect was greater for small targets than large targets (176 ± 15 ms vs. 31 ± 13 ms).

**Figure 2:**
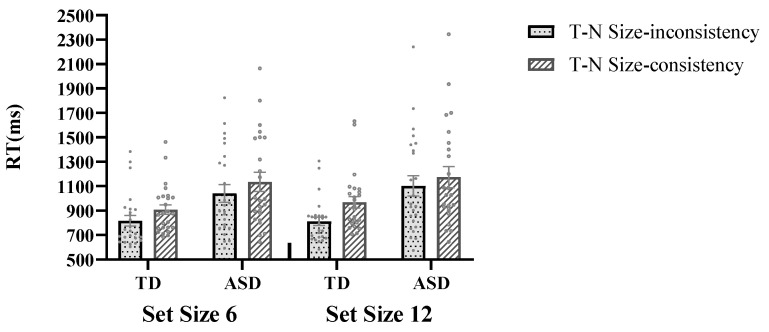
The RT differences in T-N size-consistency between ASD and TD across two set sizes: the TD group revealed a significant interaction between set size and T-N size-consistency, showing a stronger T-N size-consistency effect at the large set size compared to the small set size. Conversely, there was no significant interaction between set size and T-N size-consistency in the ASD group. Error bars denote the standard error of the mean RT.

Importantly, there was a significant interaction between group and T-N size-consistency (Figure 2), *F* (1, 47) = 6.43, *p* = 0.015, *η*^2^ = 0.12, indicating that the T-N size-consistency effect was more pronounced in TD compared to ASD (124 ± 11 ms vs. 83 ± 11 ms). Moreover, a significant three-way interaction was observed involving group, set size, and T-N size-consistency (Figure 2), *F* (1, 47) = 9.29, *p* = 0.004, *η*^2^ = 0.17. Subsequent analysis within the TD group revealed a significant interaction between set size and T-N size-consistency, *F* (1, 24) = 11.52, *p* = 0.002, *η*^2^ = 0.32, showing a stronger T-N size-consistency effect at the large set size compared to the small set size (156 ± 16 ms vs. 91 ± 15 ms). Conversely, there was no significant interaction between set size and T-N size-consistency in the ASD group, *F* (1, 23) = 1.14, *p* = 0.296, *η*^2^ = 0.05. All other interaction effects were found to be insignificant.

**Figure 3:**
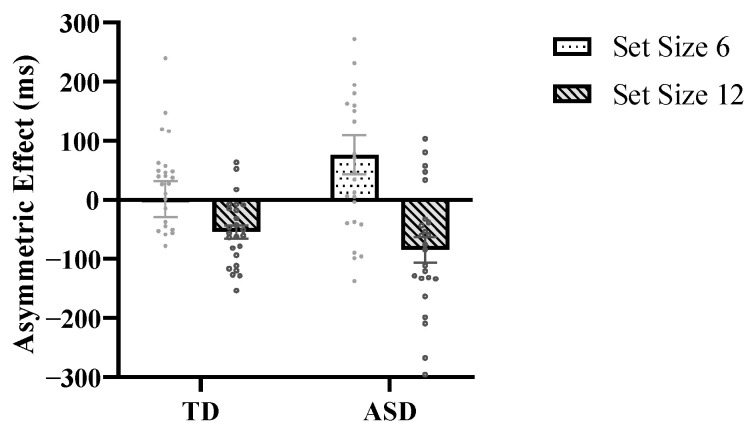
The size asymmetry effect of ASD and TD in two set sizes: the size asymmetry effect is calculated as the RT for a small target among large distractors minus the RT for a large target among small distractors. A positive value indicates faster responses in searching for a small target among large distractors; a negative value indicates faster responses in searching for a large target among small distractors. Error bars represent the standard error of the mean RT. In the TD group, there was no noteworthy contrast in the size asymmetry effect between the large and small set sizes. However, in the ASD group, the size asymmetry effect was noticeably more pronounced at the large set size compared to the small set size.

Furthermore, the T-N size-inconsistency condition in this study encompasses two search paradigms: identifying a small target within large distractors and locating a large target within small distractors. The difference between these two conditions reflects search asymmetry based on size. The size asymmetry effect is calculated as the RT for a small target in large distractors minus the RT for a large target in small distractors (RT_T-N size-consistency_ − RT_T-N size-inconsistency_ = T-N size-consistency effect). A positive value indicates faster responses when searching for a small target in large distractors, whereas a negative value suggests quicker responses for a large target in small distractors. A repeated-measures ANOVA was conducted with 2 (group: TD vs. ASD) × 2 (set size: 6 vs. 12) × 2 (target size: large vs. small), examining the size asymmetry effect (see Figure 3). The results revealed no significant main effect of group, *F* (1, 47) = 0.78, *p* = 0.382, *η*^2^ = 0.02. The main effect of set size was significant, *F* (1, 47) = 17.56, *p* < 0.001, *η*^2^ = 0.27, with the size asymmetry effect at the large set size being significantly stronger than that in the small set size (−69 ± 12 ms vs. 39 ± 23 ms). Notably, the interaction between group and set size was significant (Figure 3), *F* (1, 47) = 4.12, *p* = 0.048, *η*^2^ = 0.08, indicating that in the TD group, the size asymmetry effect did not significantly differ between large and small sets (−54 ± 17 ms, 95% CI [−88, −21] vs. 2 ± 32 ms, 95% CI [−62, 65], *p* = 0.129). In a large set size, the RT for small targets was faster (785 ± 37 ms vs. 840 ± 35 ms, *p* = 0.002), demonstrating a search advantage for larger targets. Conversely, in a small set size, the RT did not significantly differ between the two targets (818 ± 43 ms vs. 817 ± 48 ms, *p* = 0.96), indicating no size asymmetry. In the ASD group, the size asymmetry effect was significantly more pronounced at the large set size (76 ± 34 ms, 95% CI [11,141]) compared to the small set size (−85 ± 21 ms, 95% CI [−119, −51], *p* < 0.001). Specifically, at the large set size, the RT for small targets was faster than for large targets (1061 ± 81 ms vs. 1145 ± 86 ms, *p* < 0.001), whereas in the small set size, the RT for large targets was faster than for small targets (1003 ± 63 ms vs. 1079 ± 83 ms, *p* = 0.022).

## 4. Discussion

This investigation utilized a visual search paradigm to assess whether individuals with ASD demonstrate search advantage in feature search tasks and evaluate the influence of perceptual load and distractor grouping during the search process. Additionally, the study examined size asymmetry between the ASD and TD groups. The empirical data revealed that search speed was significantly slower in the ASD group than the TD group, yielding no evidence of a visual search advantage in the ASD group for this specific task. However, enhanced distractor grouping was associated with improved target detection speed in participants with ASD. Notably, at the large set size, the ASD group showed a diminished capacity for distractor grouping compared to the TD group. Furthermore, size asymmetry in the visual search in the ASD group was modulated by perceptual load. Participants with ASD detected small targets faster at the small set size and processed large targets faster at the large set size. These findings suggest that distractor grouping facilitates target detection under a small set size, whereas attentional resources are predominantly allocated to target processing under a larger set size, leading to reduced processing of distractors.

Firstly, in this study, individuals with ASD exhibited slower average search speeds in visual search tasks compared to TD, without demonstrating an advantage. Prior research has predominantly reported enhanced performance in ASD cohorts in moderately challenging conjunction search paradigms (Shirama et al., 2017), whereas no such advantage was observed in simpler feature search tasks (Keehn et al., 2013). In more demanding multi-conjunction search conditions, ASD participants exhibited slower search rates than TD (Keehn & Joseph, 2016). Consequently, some scholars posit that the advantage in individuals with ASD is modulated by task complexity (Chen et al., 2020; Schlooz & Hulstijn, 2014). Our experimental protocol incorporated multiple stimulus categories with distractors and targets randomly distributed, thereby increasing task difficulty relative to single-feature paradigms. Nonetheless, no search performance advantage was detected in ASD individuals, which may be attributable to the task difficulty remaining below that of conjunction searches or to the possibility that task complexity is not the primary determinant of ASD-related visual search performance.

Some research suggests that top-down and bottom-up attentional control and perceptual mechanisms are critical determinants of the superior visual search performance observed in ASD populations (Amrita et al., 2014; Keehn & Joseph, 2016). Specifically, Wolfe and Gancarz (1997) proposed a guided search model partitioning attentional guidance into top-down and bottom-up components. Top-down guidance involves goal-directed focus based on task objectives, whereas bottom-up guidance is driven by stimulus salience, such as local contrast or physical prominence. The magnitude of disparity among search items or the salience of the target correlates with increased search efficiency (Koehler et al., 2014; Wolfe, 2021). Keehn and Joseph (2016) employed multiple conjunction search tasks with variable set sizes, where targets were predefined but initially unknown, and distractors were specified, thereby minimizing the influence of top-down and bottom-up attentional and perceptual processes during search. They did not observe a search advantage in individuals with ASD. Similarly, our study utilized four stimulus modalities, presenting multiple search conditions with varying sizes, shapes, and randomized spatial arrangements. Targets were also unknown prior to the search, with only distractors defined, yet no specific advantage was observed in the ASD group. These findings imply that the typically observed superior performance of ASD individuals in visual search tasks may critically depend on the engagement of top-down or bottom-up attentional and perceptual mechanisms.

Subsequently, this investigation elucidates that the ASD group demonstrates accelerated visual search speed under conditions of T-N size-inconsistency relative to T-N size-consistency. In the T-N size-inconsistency, distractor stimuli differ from the target in both dimensional attributes—size and shape—whereas in the T-N size-consistency, distractors vary solely in shape but share the same size as the target. Consequently, the strength of distractor grouping is weaker in the T-N size-consistent condition compared to the inconsistent condition. When T-N size-inconsistency is present, the distractor grouping intensifies, thereby facilitating expedited search performance (Nayar et al., 2017). Our findings suggested that individuals with ASD are capable of executing distractor grouping during visual search tasks (Sabrina et al., 2023) and that augmenting the perceptual grouping strength of distractors can enhance target detection efficiency.

Additionally, the study demonstrates that under a large set size, the influence of T-N size-consistency on search performance in individuals with ASD is attenuated relative to TD individuals. This disparity may reflect fundamental differences in perceptual processing mechanisms between ASD and TD individuals (Burns et al., 2017; DuBois et al., 2017; Dellapiazza et al., 2018). Prior research generally indicates that ASD individuals possess reduced overall perceptual processing capacity and exhibit a bias toward local feature analysis (Evers et al., 2018; Kaldy et al., 2016; Manjaly et al., 2007), with the degree of perceptual specificity positively correlating with symptom severity (Gliga et al., 2015). The Weak Central Coherence theory (Happé & Frith, 2006, WCC) and the Enhanced Perceptual Functioning model (Mottron et al., 2006, EPF) offer differing explanatory frameworks for these phenomena. In WCC theory, the global processing capacity in ASD is limited or biased (Happé & Booth, 2008), suggesting that individuals with ASD are unable to integrate a broad range of stimuli or to generalize across extensive backgrounds in visual processing (Happe, 1996). The ASD individuals may have a diminished ability to form perceptual groups in the presence of interference. They require increased temporal resources to integrate local stimuli into a cohesive perceptual gestalt compared to TD individuals, with a heightened focus on local features of target stimuli (Booth & Happé, 2018; Brinkert & Remington, 2020). Differently, both heightened local perceptual abilities and intact global processing are emphasized in the EPF model (Mottron et al., 2013). Under a large set size in our study, TD participants display significantly faster search times in the T-N size-inconsistency relative to the T-N size-consistency. This suggests that, under attentional constraints, TD individuals engage in distractor grouping, thereby reducing the attentional demand imposed by distractor stimuli. Furthermore, target salience in T-N size-inconsistency can help participants search more quickly. Although ASD individuals also exhibit improved search speeds in T-N size-inconsistency, the magnitude of the T-N size-consistency effect is diminished compared to TD individuals, indicating weaker distractor grouping in the ASD group. These findings are consistent with the WCC theory, highlighting the function of distractor grouping or target salience in size/shape feature search with alphanumeric/symbolic stimuli. The EPF model may be reflected in other experimental designs different from our experiment.

Furthermore, this investigation elucidates that both ASD and TD individuals demonstrate size asymmetry in visual search paradigms. Specifically, under conditions of low perceptual load within a small set size, the RT in ASD for a small target was faster than large target. Conversely, at the large set size, ASD individuals display a reversed size asymmetry. They detected large targets faster than small targets. In contrast, TD individuals manifest this size asymmetry exclusively in a large set size. These findings imply that perceptual load modulates size asymmetry. More precisely, under a small set size, both groups possess surplus cognitive resources, resulting in rapid target detection with no significant difference between large and small targets (Shen et al., 2021). The distractor grouping enhances visual search efficiency in individuals with ASD, evidenced by faster detection of small targets among large distractors. This phenomenon may be attributable to differential activation of cognitive resources by stimuli of distinct sizes, wherein larger stimuli engage more extensive neural processing areas (Proulx, 2010; Treccani et al., 2019). Consequently, when distractors are large, perceptual grouping processes proceed more swiftly than with smaller distractors. Under high perceptual load, available cognitive resources for distractor processing are depleted in both groups, resulting in increased attentional allocation to target stimuli. When targets are large, the relative size contrast may enhance perceptual salience, rendering large targets more perceptually prominent or proximal (Dresp-Langley & Reeves, 2020), thereby facilitating faster detection. In summary, the size asymmetry in ASD suggests that distractor grouping supports target detection under low load conditions. However, under a large set size, both ASD and TD groups exhibit convergent patterns, with limited resource availability constraining distractor processing and target detection speed primarily governed by stimulus salience.

Finally, this study has several limitations. First, the target-absent condition in the experimental design only controlled for the set size and did not manipulate stimulus size. Thus, data from the target-absent condition were solely used to ensure participants’ serious engagement with the task and could not be further analyzed to compare the speed of stimulus size judgment in target-absent scenarios. Second, similar to Keehn and Joseph (2016), this study cannot distinguish whether an advantage observed in the ASD group is related to top-down or bottom-up attentional processes. Some research suggests that individuals with ASD exhibit abnormalities in top-down visual information processing, yet intact bottom-up attention may contribute to the advantage (Toshihiko et al., 2011). Future investigations should explore whether the search advantage in individuals with ASD is more dependent on bottom-up attentional mechanisms. Third, although the experimental task involved dynamic search conditions with variable target and distractor locations, it still differs from real-world dynamic or social stimuli, such as facial motion or biological motion. Notably, attentional deficits or biases in individuals with ASD may be more pronounced during social information processing (Oomen et al., 2024; Wang et al., 2023). Subsequent studies could incorporate dynamic paradigms or compare social versus non-social stimuli to further examine individuals with ASD’s perceptual grouping capabilities under different contexts. Fourth, the findings are primarily based on behavioral data, lacking neurophysiological evidence, such as eye-tracking or neuroimaging, which limits insights into the underlying neural mechanisms of perceptual grouping in ASD. Future research could employ eye-tracking (e.g., initial fixation distribution) or EEG measures (e.g., amplitude differences in N1 components) to verify whether perceptual grouping differences occur at the perceptual stage. Given that the visual search ability and characteristics of individuals with ASD have long been a focus of research, their advantage may not follow a binary “all or none” pattern (Keehn et al., 2023). A more nuanced understanding and subdivision of the visual search patterns in this population could provide theoretical and empirical support for targeted attention training and interventions aimed at leveraging their advantages. However, such attention training or intervention strategies remain hypothetical. Future research should utilize ecologically valid stimuli and dynamic scenarios to address real-world or social contexts (such as faces or biological movements).

## 5. Conclusions

The current paper made four important points. First, individuals with ASD did not exhibit a visual search advantage in this feature search task. Second, stronger distractor grouping facilitates faster target detection in individuals with ASD. Third, both ASD and TD groups show distractor grouping influenced by perceptual load; at the large set size, the effectiveness of distractor perceptual grouping is weaker in individuals with ASD compared to TD individuals. Fourth, when target and distractor sizes were inconsistent, ASD individuals displayed size asymmetry in visual search, modulated by perceptual load. At the small set size, distractor grouping facilitates faster target search in the ASD group, with quicker processing of small target-large distractors; at the large set size, both ASD and TD groups exhibit reduced distractor processing, with faster processing of searching large target-small distractors.

In conclusion, although ASD shows no search advantage, improving distractor grouping can increase their target search speed. Nevertheless, under high perceptual load, distractor grouping in individuals with ASD is weaker than in TD individuals, consistent with the weak central coherence theory. Additionally, individuals with ASD display size asymmetry that is influenced by perceptual load, with distractor grouping aiding target detection in low perceptual load and reducing distractor processing under high perceptual load.

## Figures and Tables

**Table 1:** Demographic and cognitive characteristics of ASD and TD groups (Mean ± SE).

Variable	ASD (n = 24)	TD (n = 25)	*t*	*p*
Age (years)	19.34 ± 2.44	20.36 ± 1.70	0.770	0.445
Gender (Male)	20	21	−0.062	0.951
WAIS-IV-C Score	91.21 ± 23.33	100.28 ± 7.16	1.825	0.079
Block Design	102.61 ± 20.21	99.60 ± 10.89	−0.634	0.530
General Knowledge	97.39 ± 18.82	100.60 ± 15.30	0.650	0.519
Arithmetic	85.00 ± 18.22	97.20 ± 9.80	2.854	0.007 **
Coding	88.26 ± 23.38	104.40 ± 13.25	2.908	0.006 **

Note: ** *p* < 0.01.

**Table 2:** The RT of searching target in different T-N size-consistency and set size in ASD and TD groups (M ± SE).

	Set Size 6		Set Size 12	
	ASD	TD	ASD	TD
Large target in T-N size-consistency	1082 ± 67	844 ± 66	1069 ± 57	874 ± 56
Large target in T-N size-inconsistency	1079 ± 66	818 ± 65	1061 ± 63	785 ± 62
Small target in T-N size-consistency	1190 ± 60	972 ± 59	1281 ± 86	1064 ± 84
Small target in T-N size-inconsistency	1003 ± 56	817 ± 55	1145 ± 66	840 ± 64

## Data Availability

The raw data, experiment procedure, and stimuli of this study are available via the Open Science Framework (OSF) at https://osf.io/cenrf/?view_only=c3ed7259cb3b4391b1936011cc0e0c20 (accessed on 21 August 2025).
